# Improving seedless kelp (*Saccharina japonica*) during its domestication by hybridizing gametophytes and seedling-raising from sporophytes

**DOI:** 10.1038/srep21255

**Published:** 2016-02-18

**Authors:** Xiaojie Li, Zhuangzhi Zhang, Shancun Qu, Guangjin Liang, Juan Sun, Nan Zhao, Cuiju Cui, Zengmei Cao, Yan Li, Jinhua Pan, Shenhui Yu, Qingyan Wang, Xia Li, Shiju Luo, Shaofeng Song, Li Guo, Guanpin Yang

**Affiliations:** 1 National Engineering Science Research & Development Center of Algae and Sea Cucumbers of China, Yantai, Shandong 264003, PR China; 2 Provincial Key Laboratory of Genetic Improvement & Efficient Culture of Marine Algae of Shandong, Yantai, Shandong 264003, PR China; 3 Shandong Oriental Ocean Sci-tech Co., Ltd., Yantai, Shandong 264003, PR China; 4 Laboratory of Marine Genetics and Breeding, Ocean University of China, Qingdao 266003, PR China; 5 Institutes of Evolution and Marine Biodiversity, Ocean University of China, Qingdao 266003, PR China; 6 College of Marine Life Sciences, Ocean University of China, Qingdao 266003, PR China

## Abstract

Dongfang no.7 (*Saccharina japonica*) was bred and maintained by hybridizing gametophytes, self-crossing the best individuals, selecting the best self-crossing line and seedling-raising from yearly reconstructed sporophytes. It increased the air dry yield by 43.2% in average over 2 widely farmed controls. Dongfang no.7 was seedling-raised from bulked sporophytes reconstructed from its representative gametophyte clones. Such strategy ensured it against variety contamination due to possible cross fertilization and occasional mixing and inbred depletion due to self-crossing number-limited sporophytes year after year. It derived from an intraspecific hybrid through 4 rounds of self-crossing and selection and retained a certain degree of genetic heterozygosity, thus being immune to inbred depletion due to purification of unknown detrimental alleles. Most importantly, it can be farmed in currently available system as the seedlings for large scale culture can be raised from reconstructed Dongfang no.7 sporophytes. Breeding and maintaining Dongfang no.7 provided a model that other varieties of kelp (*S. japonica*) and brown algae may follow during their domestication.

A non-flowering, non-vascular and seedless seaweed with invaluable economic and ecological importance, kelp, or Haidai in Chinese, is actually a marine crop, but not the normal one like terrestrial rice, wheat and corn. Its sporophytes are harvested as food, feed or the raw material of alginate and mannitol extraction. Chinese kelp breeders and farmers refer *Saccharina japonica* (Areschoug) CE Lane, C Mayes, LD Druehl & GW Saunders (Phaeophyceae, Laminariales)^1^,^2^ to kelp although other species, *e.g.* giant kelp (*Macrocystis pyrifera*), have also been tentatively farmed in China in recent decades^3^. They also call kelp either Japanese kelp or true kelp as its scientific name *S. japonica* indicated; unfortunately it may have been contaminated genetically by *S. longissima* as two interspecific hybrids^4^,^5^ and an interspecific hybrid derived variety^6^ at least have been bred and commercially farmed in recent years. Such contamination has been evidenced recently by massively parallel sequencing^7^. Kelp has contributed significantly to the mariculture industry of China; its farming area has reached ~37 thousand hectares and its air dry yield has reached about one million ton in 2013 as were documented in the Annual Report of Chinese Fisheries 2014. Except for mannitol and alginate extraction, kelp promises also to be one of most favorite feedstock for bioethanol fermentation as its three major components, mannitol, glucan and alginate, can be fermented into bioethanol concertedly^8^,^9^,^10^.

As mosses and ferns do, kelp performs an alternative life cycle during which large and multicellular sporophytes alter with microscopic and less cellular gametophytes. The sporophytes produce motile zoospores which develop into male and female gametophytes. The gametophytes produce sperms and eggs, respectively, which fuse to form zygotes. The zygotes subsequently develop into sporophytes. Under artificial conditions, kelp gametophytes can be asexually cloned and hybridized (Supplementary Fig. 1)^11^,^12^,^13^,^14^, making breeding and seedling-raising kelp *elite* varieties feasible by hybridizing these clones^4^,^5^,^6^,^15^,^16^,^17^.

During its domestication and farming for nearly a century in China, kelp has received an intensive genetic improvement, and a set of *elite* varieties have been bred and commercially farmed^*e*.*g*.,^^18^,^19^, which have increased yield by a wide margin (~20 ton.ha.^−1^y^−1^) (~30% of the theoretical 59 ton.ha^−1^y^−1^)^10^. At least two intraspecific hybrids of *S. japonica* have been bred but not tentatively farmed early^15^,^16^. Recently, a variety, 90-1, was bred and commercially farmed by making an interspecific hybrid of *S. japonica* and *S. longissima* and then self-crossing and selecting continuously^6^. Variety 90-1 increased the yield to ~30 ton.ha.^−1^y^−1^ (>50% of the theoretical); however it was seedling-raised from bulked sporophytes matured year after year. Such mode of seedling-raising from number-limited sporophytes may meet species mixing, cross fertilization and inbred depletion as were observed in practice. In order to avoid these entanglements, 2 interspecific (Dongfang no. 2 and 3) and one intraspecific (Dongfang no.6)^17^ hybrids have been bred following a strategy very effective for rice^20^, *i*.*e*. direct utilization of heterosis (or hybrid vigor)^4^,^5^. The hybrids were seedling-raised by crossing parental gametophytes with their biomass amplified ahead. Interspecific hybrids dramatically increased the yield by>30% over that of traditional varieties. They avoided also the disadvantages of seedling-raising from sporophytes^21^,^22^; however, the methodological complexity and facility specificity of seedling-raising these hybrids made their farming in current system difficult.

Processing and consumption of kelp greatly appreciate wide and smooth (suitable for processing flake and stripe products) and dark brown (critical for air drying quality) blades. In breeding practice of kelp hybrids and hybrid derived kelp varieties^4^,^5^,^6^,^17^, trait complementarity between either parental sporophytes or the source sporophytes of parental gametophytes has been found to be common. Aiming to develop a kelp variety with the characteristics of wide and smooth and dark brown blades, the compatibility with current seedling-raising system, and the immunity to variety contamination and inbred depletion, Dongfang no.7 was bred and maintained by hybridizing the gametophytes isolated from two sporophytes with complementary morphological traits, self-crossing the best individual and selecting the best self-crossing line, and seedling-raising from the sporophytes of the bred variety reconstructed from its representative gametophytes.

## Materials and Methods

### Isolation and preservation of gametophyte clones

Isolation and preservation of gametophyte clones were carried out following the methods we described early^5^,^23^. In brief, a tissue block with sporangia was cut out of a mature sporophyte, cleaned thoroughly, submerged in 1.5% KI and air-dried briefly. The block was submerged in seawater again to release zoospores which adhered to the bottom of a Petri dish, and germinated there. The young gametophytes were picked up when their gender was distinguishable (the female was larger and more cellular while the male was smaller and less cellular), transferred into NaNO_3_ and KH_2_PO_4_ enriched seawater, cultured to a desirable amount of biomass, and preserved in NaNO_3_ and KH_2_PO_4_ enriched seawater at low temperature and under weak irradiation. The gender of gametophyte clones was further verifiable during their biomass amplification and hybridization.

### Gametophyte clone hybridization and hybrid seedling-raising

As were done for Dongfang no. 2^5^, the gametophyte clones were biomass amplified, filtration collected and weighed, mixed, and homogenized. The homogenate was cultured under a short-day regime, homogenized again when the egg dropping was observable, filtered, and sprayed onto coir rope curtains. The gametophytes adhered to the curtain and zygotes developed into young sporophytes there. The hybrid was seedling-raised indoor, cultured temporarily in sea when they were 1.5–2.0 cm in length to approximately 20 cm, and then inserted in between the skeins of hanging ropes arrayed between two headropes which were floated by buoys and anchored to sea bottom (floating nets rather than floating rafts although it was called such) (http://www.fao.org/docrep/field/003/AB724E/AB724E00.htm) (Supplementary Fig. 2).

### Seedling-raising from bulked sporophytes

The seedlings of Dongfang no. 7 and 2 controls were raised with traditional summer seedling-raising from bulked sporophytes method^21^,^22^which has been optimized for current farming system. A collection of mature sporophytes were intensively washed and briefly air dried, which were then sunk into seawater, letting zoospores release there. After cleaning, the coir rope curtains were sunk into zoospores-containing seawater, to them zoospores adhere. The curtains were arrayed at the bottom of cement pools where gametophytes develop, yielding eggs and sperms. The sperms fertilized eggs attaching to female gametophytes, forming zygotes which develop into young sporophytes. In order to delete weak sporophytes and unwanted organisms (*e.g.* microalgae), the curtains were washed with high pressure seawater frequently. The seedlings were cultured indoor in flowing cool seawater (8 °C) over the whole summer (from the last 10 days of July to the first 10 days of October, about 3 months), temporarily cultured in open sea when seawater temperature was < 20 °C ,transplanted by inserting seedlings in between the skeins of hanging ropes, cultured in open sea and harvested finally (Supplementary Fig. 3).

### Genotyping gametophyte clones

A set of female gametophyte clones and a set of male ones were selected following a philosophy that the characteristics of the source parental sporophytes may complementarily dominate their hybrid^4^,^5^,^6^,^17^ from the core gametophyte clone collection of kelp germplasm maintained in weak light and at low temperature by National Engineering Science Research & Development Center of Algae and Sea Cucumbers of China; Provincial Key Laboratory of Genetic Improvement & Efficient Culture of Marine Algae of Shandong, Shandong Oriental Ocean Sci-tech Co., Ltd., Yantai, Shandong, China, which were biomass amplified on small scales with the method we described early^5^, harvested by filtrating through a sieve cloth and stored in 70% ethanol-15 mM EDTA (pH 8.0) and at −20 °C.

As we found early, the heterosis (hybrid vigor) of a hybrid is proportional to the genetic distance between its parental gametophyte clones to a certain extent^24^. In order to reduce the number of possible crossing combinations, the selected clones were genotyped at 23 microsatellite loci (Supplementary Table 1)^25^,^26^,^27^. DNA was isolated from about 100 mg gametophyte following Li et al. (2007)^5^. A PCR reaction was performed in a 25 μL volume containing 1 × reaction buffer, 2.0 mM MgCl_2_, 200 μM dNTP (each), 200 μM primers (each direction) and 1U *Taq* DNA polymerase. The reaction was thermocycled by pre-denaturing at 94 °C for 4 min, followed by 30 cycles of denaturing at 94 °C for 1 min, annealing at the temperature appropriate for primer pairs each (Supplementary Table 1) and extending at 72 °C for 1 min and a final extension at 72 °C for 10 min. PCR product was separated on 6% denaturing polyacrylamide gel and visualized by silver staining^28^. Sporophytes were genotyped as were done for gametophyte clones.

### Determining the growth performance

Twenty four blades, 6 each hanging rope, 2 at upper, middle and lower positions, respectively, 4 ropes each variety, were selected, labeled and measured for daily growth and daily tissue loss of blade in the whole culture season following the strategy adopted for Dongfang no. 2, 3 and 6^4^,^5^,^17^. The blade fresh weight, blade dry weight and ratio of fresh to dry weight were also measured. In total, 24 blades were selected as were done for daily growth and daily tissue loss of blade, which were sampled each time with their weight, length, width and thickness measured independently. Blade length was the distance between the base and the tip of blade. Blade width was the maximum of blade. Discs were taken at 3 of the 5 points (very tip and very base were excluded) that divide a blade into 4 equal segments. Three discs were piled up with the total thickness averaged as the thickness of blade. These blades (with the discs for thickness measurement) each were then air dried completely and weighed as blade dry weight. The ratio of fresh to dry weight was calculated by dividing fresh weight with dry weight.

### Morphological characterization at harvesting

Once being harvested, 50 blades were randomly selected with their length, width and thickness measured independently. These blades were bulked and weighed with the total averaged as blade fresh weight, and then air dried to completion and weighed with the total averaged as blade dry weight, which avoided the inconvenience of independent measurement but lost the chance of calculating standard errors.

### Reconstructing Dongfang no. 7 and 2 source varieties from gametophytes

Dongfang no. 7 was reconstructed from the gametophyte clones isolated when it was bred. These gametophyte clones were preserved as its representative germplasm. Except for amplifying the biomass independently and mixing at equal amounts, the seedling-raising was carried out as for hybrids^5^. This strategy was followed in seedling-raising Kuanbao haidai and Korean kelp, 2 source varieties from them 2 parental gametophytes of Dongfang no.7 were cloned.

### Measuring temperature and transparency of surface sea water

The surface seawater temperature was measured with a thermometer which was sunk into seawater 0.5 m apart from vessel and 1 meter in depth for at least 10 min ahead of reading. Temperature was read 3 times, 3 min in seawater and reading quickly each time. Three reads were averaged as the measured. The seawater transparency was measured with a white metal disc, 30 cm in diameter and a 5 kg lead plate attached to bottom. The disc was sunk into seawater without direct sun shine till the disc was not seeable with naked eyes with the depth scale on hanging rope read each time. The reads of 3 persons, 3 each, were averaged as the transparency.

### Data processing

If applicable, observations were expressed as mean ± SD. The significant difference among a group of means was determined through ANOVA with SPSSv.17.0 (http://softadvice.informer.com/Spss_Statistics_17.0_Free_Download.html). The significant difference was accepted if *p* ≤ 0.05. The morphological similarity of a trait was also described with variation index defined by Zhang et al. (2007)^6^, *i*.*e*. standard error/ mean. The Nei’s original measure of genetic distance^29^ of a gametophyte combination and the genetic parameters (*Na*, *Ne*, *He*, *Ho*, *H* among others) were calculated using Popgene 32 (https://www.ualberta.ca/~fyeh/popgene_download.html) and Genepop (http://genepop.curtin.edu.au/). In kelp farming practice, the air dry yield was deduced from air dry weight of blade. The number of blade per hectare was assumed to be 180,000, thus estimated yield per hectare = blade dry weight × 180,000. Yield increase = [(the yield of Dongfang no. 7 − that of control)/that of control] × 100.

## Results

### Breeding and characterizing Dongfang no.7

A set of female and male gametophyte clones were genotyped at 23 microsatellite loci (Supplementary Table 1). The genetic distance between gametophytes ranged from 0.693 to 1.540. In total, 22 combinations varying in genetic distances between 1.010 and 1.540 were crossed. Trait comparison targeted one hybrid of a female gametophyte clone from Kuanbao haidai and a male one from Korean kelp, both belong to *S. japonica*.

**Figure 1:**
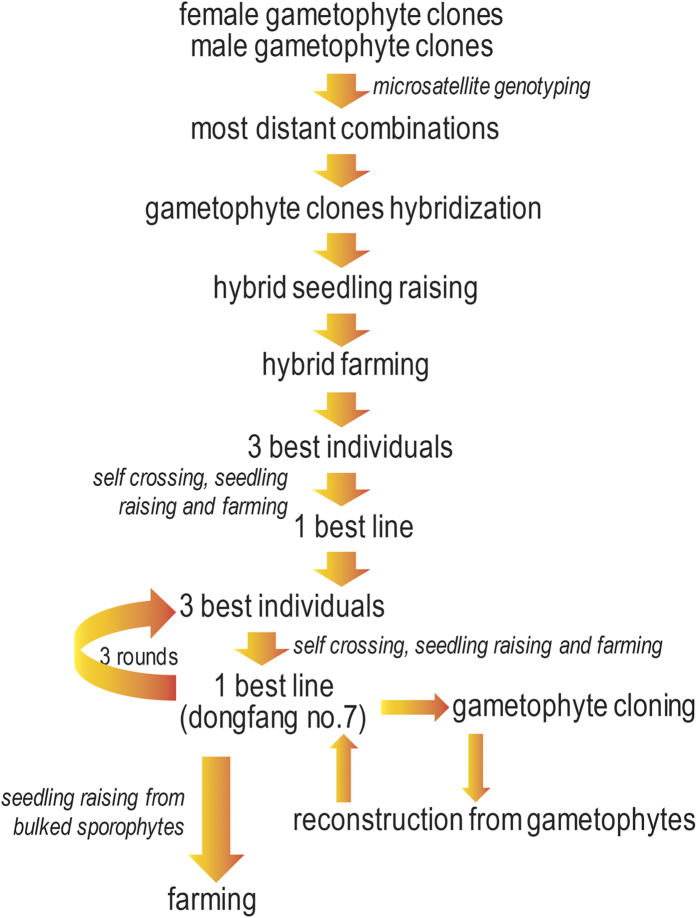
Flow chart of breeding and maintaining Dongfang no.7 (*S. japonica*).

**Figure 2:**
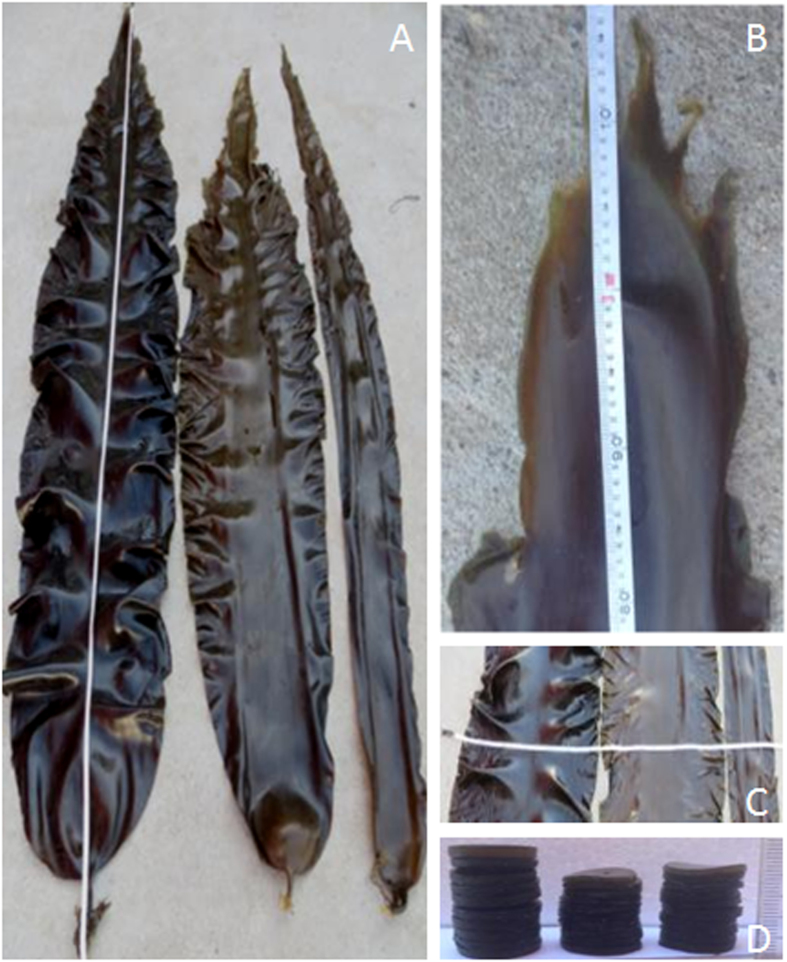
The morphological characteristics of Dongfang no. 7. (**A**), the blades of Dongfang no. 7 (below a ruler, left), Kuanbao haidai (middle) and Korean kelp (right); (**B**), the blade tip of Dongfang no. 7 under a ruler; (**C**), the widest part of Dongfang no. 7 (left), Kuanbao haidai (middle) and Korean kelp (right) under a ruler; (**D**), Blade disc pile, 10 each, of Dongfang no. 7 (left), Kuanbao haidai (middle) and Korean kelp (right) beside a ruler.

**Figure 3:**
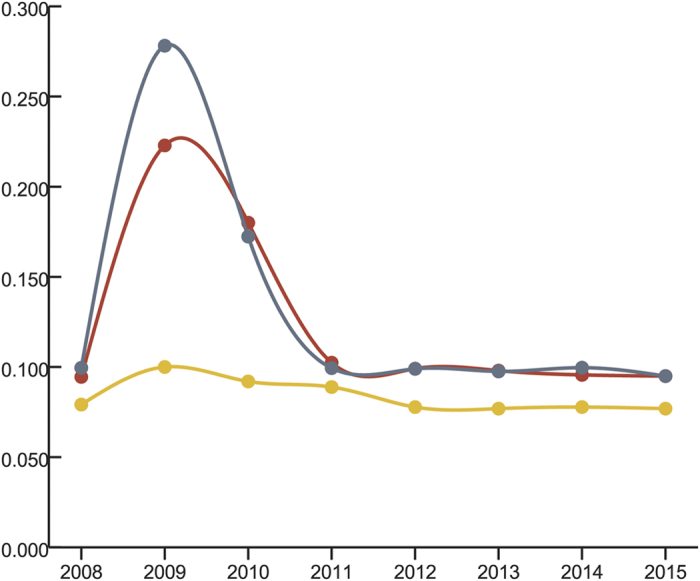
Change of the variation index of blade length (red), width (grey) and thickness (yellow) during breeding.

Four rounds of self-crossing 3 best sporophytes and selecting one best self-crossing line generated Dongfang no.7 (Fig.1) which was characteristic of wide, thick, smooth, dark brown and wrinkle less blade, strong holdfast, flat-cylindrical stripe, oblate base, non-obvious vertical central groove, and less margin (Fig. 2). The traits of Dongfang no.7 fluctuated during breeding and culturing by seedling-raising from sporophytes matured year after year (Table 1, Supplementary Fig. 4); however the variation index of blade length, width and thickness indicated a fast stabilization of morphological traits in 4 rounds of self-crossing 3 best individuals and selecting the best self-crossing line (Table 1, Fig. 3). Air dry weight of blade and yield fluctuated also yearly; however the heterosis may have drowned out such fluctuation during breeding. Self-crossing the best individuals and selecting the best self-crossing line even made the heterotic performance of these 2 traits better than that of hybrid itself (Table 1, Supplementary Fig. 4).

The hybrid showed obvious heterosis in air dry blade weight over two source sporophytes of its parental gametophytes (35.5%). The maximum increase of air dry blade weight observed during breeding (2008–2012) reached 45.0% over 2 source sporophytes. Dongfang no.7 increased the yield by 43.2% over 2 controls in average (Table 2). The genetic parameters (*Na*, *He* and *H*) of Dongfang no.7 were obviously lower than those of two controls (Table 3).

### Growth performance

The maximum daily growth was similar among Dongfang no.7, 2 controls and 2 source varieties of parental gametophytes; however Dongfang no.7 grew faster than others on most days including those when seawater temperature and transparency were the lowest. When seawater temperature was > 6 °C, Dongfang no.7 slowed down its growth less obviously than others. Dongfang no. 7 maintained its growth till seawater temperature was < 14.8 °C, higher than others did (Supplementary Fig. 5a). Dongfang no. 7 lost less tissue of blade than 2 controls and 2 source varieties of parental gametophytes. Such difference was pronounced when seawater temperature was > 13 °C (Supplementary Fig. 5b). The total loss of blade tissue of Dongfang no. 7 was 96.5 cm, significantly shorter than that of two controls (161.7 cm and 134.5 cm, respectively, *p* < 0.01), but slightly higher than that of Kuanbao haidai (93.2 cm) and Korean kelp (87.2 cm). Dongfang no.7 lost 26.0% of the total blade length, shorter than that of others did (27.9–50.1%).

The blade length of Dongfang no.7, 2 controls and 2 source varieties of parental gametophytes reached their maximum length synchronically (Supplementary Fig. 6a) but the blade width and thickness did differentially. Dongfang no.7 reached the maximum blade width synchronically with Kuanbao haidai but later than 2 controls and Korean kelp (Supplementary Fig. 6b). It reached the maximum blade thickness later than Control 2 and Kuanbao haidai, but earlier than Korean kelp (Supplementary Fig. 6c). Dongfang no.7 grew to the maximum fresh weight synchronically with Control 1 but did so later than others (Supplementary Fig. 7a). It accumulated the maximum amount of dry materials almost synchronically with Control 1 but later than others (Supplementary Fig. 7b). A ratio of < 8.3: 1 (fresh: dry weight) was empirically believed to be suitable for harvesting. Dongfang no. 7 touched such ratio later than Control 1 and Korean kelp but earlier than Control 2 and Kuanbao haidai (Supplementary Fig. 7c). Dongfang no. 7 reached such ratio when seawater temperature was 12.5 °C, similar to that of Control 1 but lower than that of Control 2. These characteristics provided clues to that Dongfang no.7 was of a moderate harvesting time.

### Maintaining Dongfang no.7

The trait difference between Dongfang no.7 seedling-raised from sporophytes matured year after year (DF7^*^, see Table 1 for these abbrs.) and the reconstructed from gametophyte clones (rDF7) varied between 0.00% and 1.33% of rDF7 with an average of 0.81% as were observed in 2014. The trait difference of the reconstructed Dongfang no.7 (rDF7) from the seedling-raised from reconstructed sporophytes (DF7^**^) was less so, varying between 0.00 and 2.43% of DF7^**^ with an average of 1.13% as were documented in 2015; however the traits of both rDF7 and DF7^**^ were different from those of the seedling-raised from sporophytes matured year after year (DF7^*^) either significantly (blade length and width (*p* < 0.05) or obviously (blade fresh weight, blade dry weight and yield) (Table 1). These findings indicated that seedling-raising from number limited sporophytes matured year after year has caused inbred depletion which can be avoided by reconstructing sporophytes from gametophytes. The 40 gametophyte clones representing Dongfang no. 7, 20 each gender, carried a similar number of alleles per locus (1.810 *vs*. 1.826). The reconstructed Dongfang no. 7 inherited the same number of alleles as that of gametophyte clones (1.810) (Table 3).

## Discussion

Summer seedling-raising from bulked sporophytes matured year after year shares the cool seawater in current seedling raising system of China. Sperms of a variety may cross fertilize the eggs of the others although such possibility was not evidenced yet. Occasional mixing may take place during seedling-raising. Such mode of seedling-raising from number-limited sporophytes mature year after year may also cause inbred depletion which is extremely obvious as kelp is on its way of domestication. Seedling-raising from gametophytes as was done for kelp hybrids may avoid these disadvantages; unfortunately methodologically complex and facility specificity made such strategy incompatible with the system in use.

Breeding and maintaining Dongfang no.7 provided a model that the genetic improvement of seedless kelp (*S. japonica*) and other brown algae may follow. It was bred by crossing gametophytes and self-crossing the best individuals and selecting the best self-crossing line. Its sporophytes were reconstructed each year from representative gametophyte clones, from which seedlings were raised for farming. Such strategy ensured Dongfang no.7 against variety contamination due to cross fertilization and occasional mixing and inbred depletion due to self-crossing number-limited sporophytes matured year after year. Dongfang no.7 derived from an intraspecific hybrid through 4 rounds of self-crossing and selection, and retained a certain degree of genetic heterozygosity, thus being immune to inbred depletion due to diversity reduction. Most importantly, farming Dongfang no.7 was compatible with in use farming system. It increased the air dry yield by 43.2% over 2 widely farmed controls in average, close to the increased of intraspecific hybrid^17^ but less than that of interspecific hybrids or the varieties derived from them^4^,^5^,^6^. Such strategy was feasible at least for genetically improving the brown algae with a similar life cycle, *e*.*g*., *Undaria pinnatifida* and *M. pyrifera*.

Self-crossing best sporophytes and selecting best self-crossing line should have purified the loci associating with morphological traits; however those without selection may have retained a high heterozygosity, which may aid to avoiding inbred depletion. Diverse hypotheses including dominance (complementation), overdominance and epistasis, have been proposed to understand the genetic mechanism of heterosis (hybrid vigor, heterotic performance)^28^; however these hypotheses based on the performance of hybrids and their parents. Being different from hybridization and selection widely practiced in terrestrial crop breeding, we have traced the performance of the best line generated by self-crossing the best individual for 4 rounds. It was interesting to note that the best self-crossing line of round 2 through 4 performed better than hybrid itself in air dry weight of blade and yield deduced from it. We preferred to explain the heterosis with “the accumulation of numerous rare superior alleles with positive dominance”^30^; such mode of breeding may have retained superior alleles with positive dominance but eliminated inferior alleles at loci associating with morphological traits. Unfortunately, some superior alleles may get lost during multiple rounds of selection and culture of the seedling raised from sporophytes matured year after year. Our findings indicated that a pure variety comparable with and even better than hybrid itself could be developed by self-crossing the best individuals and selecting the best self-crossing line.

Early QTLs mapping trials have used diverse populations like F_2_, backcross inbred lines (BILs), recombinant inbred lines (RILs) and double haploid lines (DH). Unfortunately, either non-repeatable observations (F_2_) or interactions among various chromosomal segments (BILs, RILs and DH) hindered their application to fine mapping and map-based cloning. Near-isogenic lines (NILs) are uniquely advantageous to QTLs mapping; QTLs may be assigned to a chromosomal segment without any interference. Each NIL carries either one or a few chromosomal segments of donor parent in the background of recurrent one. Once these chromosomal segments become traceable or are defined by either molecular markers or DNA sequence, NILs evolve into chromosome segment substitution lines (CSSLs), which cover the entire donor genome in combination. Secondary F_2_ can be generated by backcrossing a selected CSSL with recurrent parent, and then used for fine mapping and positional cloning^31^,^32^.

Although being extremely time consuming and labor intensive, kelp NILs can be developed with currently available techniques, *i*.*e*., gametophyte cloning and hybridization^4^,^5^. The genomes of *S. japonica* and *Ectocarpus siliculosus* have been sequenced^7^,^33^. By referring to them, NILs can be genotyped by resequencing genomes either completely or partially^23^. Such trial will make QTLs mapping and even map-based gene(s) cloning of brown algae (*e*.*g*. *S. japonica* and *Ectocarpus siliculosus*) comparable with those of crops and plants.

Very rich light and heat resources in summer are wasted; kelp is not able to survive summer season. The best way out such distress is to breed high temperature tolerant varieties. We are mutating kelp zoospores, gametophytes and gametophyte fragments trying to create high temperature resistant germplasm. An alternative way toward such target is to clone gametophytes from the secondary wild sporophytes which grow weakly but survive summer seawater. They are secondary wild; they may originate from the cultured which have adapted to summer seawater in almost a century of culture in China. In order to maximize the utilization of light, heat and facility, *Gracilaria lemaneiformis* rotates with kelp on the same facility in north China. At least, farmers harvest twice a year. Many other species are tentatively cultured in recent years in order to widen the application of macroalgae and enrich the farmers. Animal culture is also highly developed in China. Unfortunately, such advance has caused diverse problems like the eutrophication of coastal waters. Following the philosophy of so-called healthy culture, kelp has been introduced into animal culturing system. Kelp biomass can be fermented into bioethanol. Such potential will certainly prompt the application of kelp to bioremediation and healthy culture of animals.

## Additional Information

**How to cite this article**: Li, X. *et al*. Improving seedless kelp (*Saccharina japonica*) during its domestication by hybridizing gametophytes and seedling-raising from sporophytes. *Sci. Rep.* **6**, 21255; doi: 10.1038/srep21255 (2016).

## Supplementary Material

Supplementary Information

## Figures and Tables

**Table 1:** The morphological characteristics of different varieties at harvesting.

Year	Line	Length (cm)	Width (cm)	Thickness (mm)	Fresh weight (kg)***	Dry weight (kg)***	Yield (ton/ha)****
	Kuanbao haidai	283.0	43.0	2.0	1.428	0.198	na
	Korean kelp	282.0	28.0	2.4	0.750	0.115	na
2008	Hybrid	333.4 ± 31.5	43.2 ± 4.3	2.4 ± 0.19	1.638	0.212	38.2
2009	SP1	315.0 ± 70.2	45.3 ± 12.6	2.4 ± 0.24	1.602	0.205	36.9
2010	SP2	335.0 ± 60.3	47.0 ± 8.1	2.5 ± 0.23	1.688	0.218	39.2
2011	SP3	326.3 ± 33.4	49.3 ± 4.9	2.7 ± 0.24	1.738	0.227	40.9
2012	SP4(DF7)	318.9 ± 31.6	48.5 ± 4.8	2.7 ± 0.21	1.710	0.221	39.8
2013	DF7*	308.2 ± 30.2	45.1 ± 4.4	2.6 ± 0.20	1.605	0.201	36.2
2014	DF7*	332.5 ± 31.8	53.2 ± 5.3	2.7 ± 0.21	1.786	0.210	37.8
	rDF7	336.5 ± 31.7	52.5 ± 5.0	2.7 ± 0.20	1.792	0.212	38.2
2015	DF7*	307.5 ± 29.2	45.3 ± 4.3	2.6 ± 0.21	1.645	0.192	34.6
	C1	286.8 ± 43.4	38.7 ± 6.4	2.4 ± 0.41	1.314	0.148	26.6
	C2	270.3 ± 47.1	39.3 ± 7.1	2.3 ± 0.47	1.310	0.144	25.9
	DF7**	327.5 ± 32.3	49.4 ± 4.8	2.6 ± 0.20	1.746	0.209	37.6
	rDF7	328.7 ± 30.6	50.6 ± 4.8	2.6 ± 0.19	1.781	0.211	38.0

^*^seedling was raised from bulked sporophytes of DF7 matured year after year.

^**^seedling was raised from rDF7 matured in 2014.

^***^the average of 50 blades.

^****^dry weight × 180.000; SP, selection population; DF7, Dongfang no. 7; C1, Control 1; C2, Control 2; rDF7, reconstructed Dongfang no. 7; na, not applicable.

**Table 2:** Observed heterosis or increase of the traits of Dongfang no.7 (%).

	Average heterosis of hybrid^1^	Maximum average increase over 2 source sporophytes	Increase of Dongfang no.7 over Control 1 2	Increase of Dongfang no.7 over Control 2 2	Average increase over 2 controls^2^
Length	18.0	18.6	14.2	21.2	17.6
Width	21.7	38.9	27.6	25.7	26.7
Thickness	9.1	22.7	8.3	13.0	10.6
Fresh weight	50.4	59.6	32.9	33.3	33.1
Air dry weight	35.5	45.0	41.2	45.1	43.2
Yield	na	na	41.4	45.2	43.2

^1^based on the traits of hybrid evaluated in 2008 and those of the source sporophyte individuals of parental gametophyte clones.

^2^based on the traits of Dongfang no.7 seedling-raised from reconstructed sporophytes evaluated in 2015; na, not applicable.

**Table 3:** The genetic parameters estimated with microsatellites.

Variety	Na	Ne	Ho	He	H
Dongfang no. 7*	1.826	1.137	0.127	0.118	0.117
Control 1	2.256	1.667	0.445	0.328	0.397
Control 2	2.135	1.568	0.419	0.307	0.385
Reconstructed Dongfang no. 7	1.810	1.162	0.141	0.134	0.142
Gametophyte clones for reconstruction**	1.810	1.122	—	—	0.160

^*^seedling-raised from bulked sporophytes of Dongfang no. 7 matured year after year.

^**^monoploidy gametophytes were treated as pure diploid; --, no biological meaning.
