# Supplementary material for: Improving seedless kelp (Saccharina japonica) during its domestication by hybridizing gametophytes and seedling-raising from sporophytes
Source: Sci Rep. 2016 Feb 18;6:21255. doi: 10.1038/srep21255 (PMC4757934; doi:10.1038/srep21255)
Supplement: Supplementary Information [file srep21255-s1.pdf]

Improving seedless kelp (*Saccharina japonica*) during its domestication by hybridizing gametophytes and seedling-raising from sporophytes

Xiaojie Li, Zhuangzhi Zhang, Shancun Qu, Guangjin Liang, Juan Sun, Nan Zhao, Cuiju Cui, Zengmei Cao, Yan Li, Jinhua Pan, Shenhui Yu, Qingyan Wang, Xia Li, Shiju Luo, Shaofeng Song, Li Guo, Guanpin Yang

Supplementary Table 1 Microsatellites used to predicting heterosis

Locus	Acc. no.	Sequence (5'-3')	*Ta* (℃)	Source
D3	CX943083	F: GCGTGGGAGGCTTTCTTC	56	29
		R: CGTGGGATTGCTGATACTG		
D5	CX943061	F: CGGCTTCATCTCCCACAG	53	29
		R: TGACGGAATAGACCCAAA		
D9	CX942859	F: CAATAATCGCAGAAAGGG	54	29
		R: CCGTCCGAACAACCAACT		
H1	DQ978338	F: CAACTCAACTACTGCCACCTA	59	30
		R: GTCCTCATCCGTTTCGTC		
H10	DQ978340	F: TATCCCGTTCGTTCCACTC	57	30
		R: CGACCCTAATAAGCTCTACCT		
H49	DQ978343	F: TGGGTATGATGGATGTCGC	59	30
		R: CAATAATAGCATGGCCGTAA		
H123	DQ978346	F: AAAGGTCGATAAGCTCGCAGTT	59	30
		R: GCGTTGTTTCGCAAAGTGATT		
Zspj5	AB859681	F: CTGTGTATGTGGAGACTGCG	62	31
		R: CTTCTGTGCTGCTGTACGTG		
Zspj6	AB859682	F: CACACCAAAATGCTGCCCTG	62	31
		R: TAGTGGGGAGGAGGGAAAAG		
Zspj8	AB859683	F: GATGGTGATATCATGGTGCG	55	31
		R: CGGCATGAACGTATGTATGG		
Zspj9	AB859684	F: GCGGCTTACCCAATGTTC	55	31
		R: ACAAACGGGGCTTCCATC		
Zspj14	AB859685	F: CTCGCTGCCACCATACATTG	55	31
		R: CGCTGTTCTACCACACGAAC		
Zspj19	AB859687	F: GCCCACCGACAACCTAATC	62	31
		R: CTGTAGTAGAGCCCATACCCC		
Zspj21	AB859689	F: CAAGTAGCAGACAACACCCC	62	31
		R: CCGAAACCTGCCGAAACT		
Zspj22	AB859690	F: TAGGACGGTATGTGTGCC	62	31
		R: GTACAGAGTAGAAGGGTGGG		
Zspj23	AB859691	F: CGAGAGACCAACACACCAAG	55	31
		R: CGAAGCTCTCCGATCATCAG		
Zspj26	AB859693	F: CGTTTGTCATCTCCCAGTTCC	62	31
		R: CCAATTACCGAGCCAAAGGAG		
Zspj28	AB859694	F: TGCTCCTACAGACGAAAGC	62	31
		R; CCCACATCTTCATCGTTGTCC		
Zspj39	AB859701	F: CTCGGTTCAAAGTTCCGCAAG	55	31
		R: CATCCGCAATTTCTTCCACGG		
Zspj40	AB859702	F: CTACCAGGCATCACACACGA	62	31
		R: GATGCGAGGGTCTATGGTCA		
Zspj43	AB859703	F: CTCGCTAGTATTCCAGAGCAG	62	31
		R: CTGTCCTTACCCAACACGAAG		
Zspj82	AB921195	F: GAGACACACTTTCAGAGGAG	58	31
		R: CTTTGTCTTCCCTTGATCCC		
Zspj89	AB921199	F: GGACAGCAATCGGTACATG	58	31
		R: GGTGAGTGAGCAACAACAG		

Acc. no., accession number; *Ta*, annealing temperature.

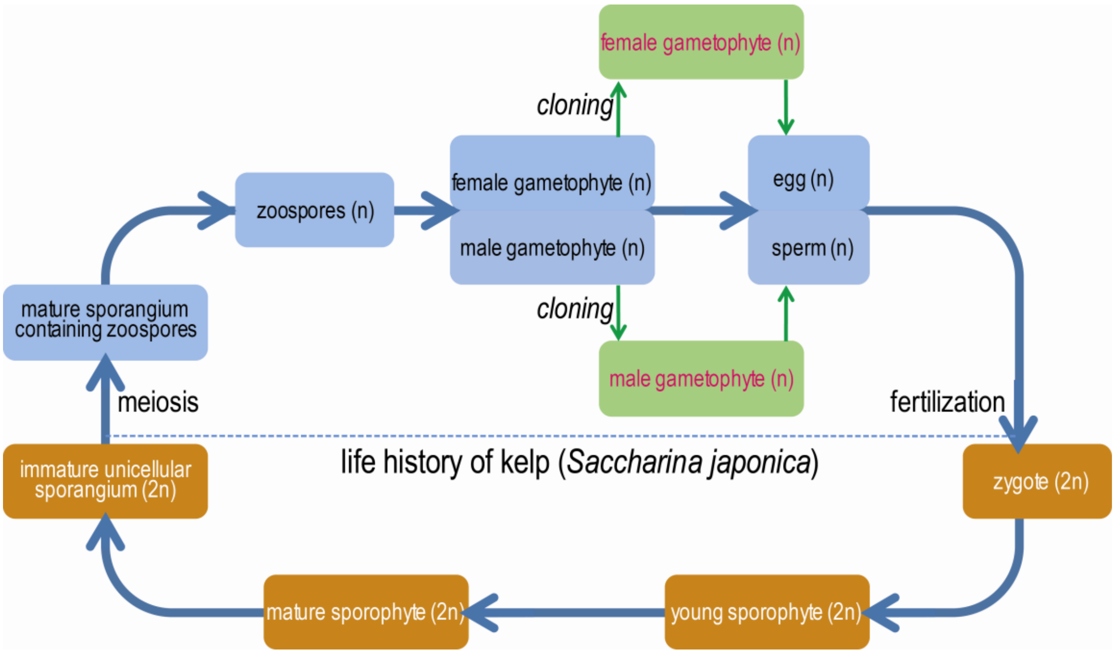

Supplementary Figure 1 Life history of kelp (*Saccharina japonica*)

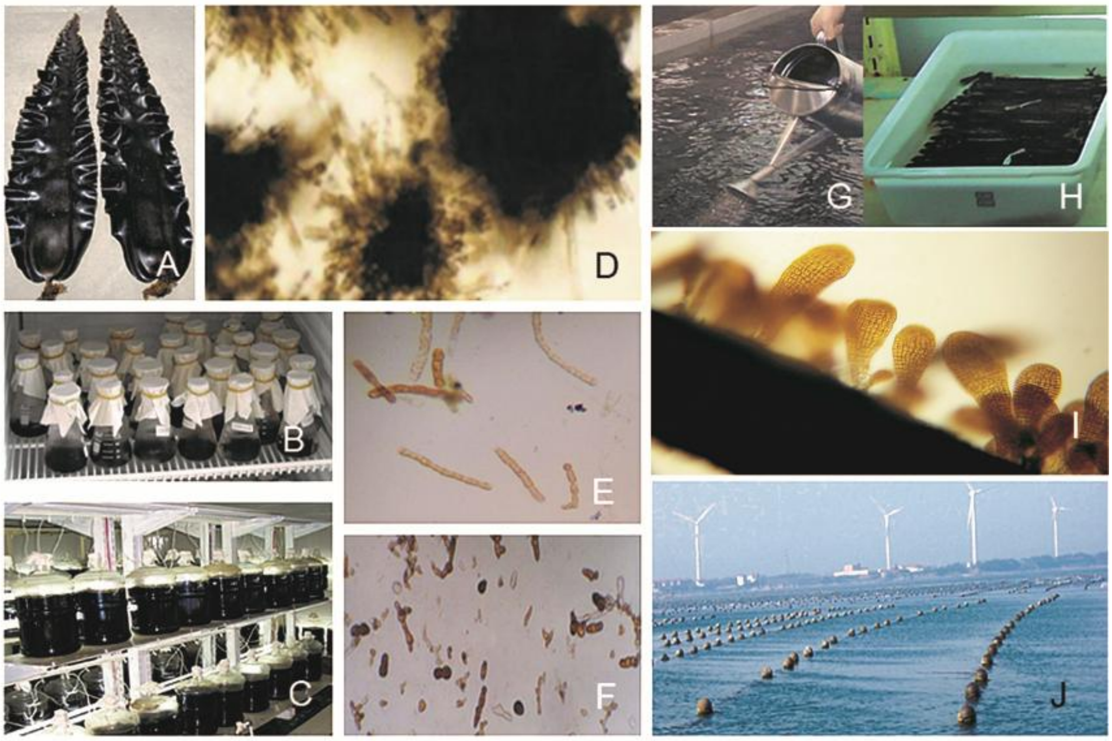

Supplementary Figure 2 Gametophyte clone hybridization and hybrid seedling-raising. From mature sporophytes (A), gametophytes are isolated, cloned and stored at low temperature and under weak light (B). Gametophyte clones are biomass amplified indoor (C), during which the gametophytes are fragmented (E) when they grow into balls (D). Such amplification may be

cycled depending on the biomass amount needed. Female and male clones are mixed, cultured and fragmented once egg releasing is observable (F). The fragmented are sprayed onto coir rope curtains in either cement pools (G) or trays (H) depending on the number of seedlings needed, on which young sporophytes developed (I). When seawater temperature is <20℃, the curtain rope is stretched out and cultured in open sea temporarily till young sporophytes are about 20cm in length. The seedlings are insertion fixed on floating ropes, cultured and harvested finally (J).

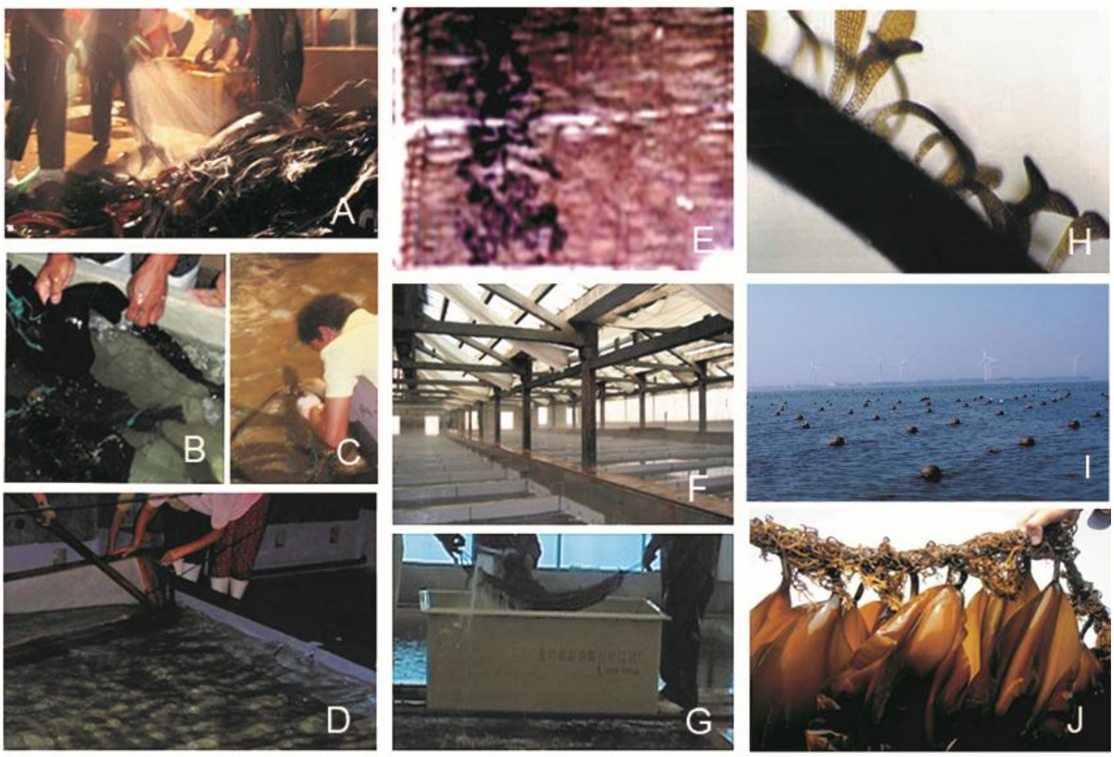

Supplementary Figure 3 Seedling-raising from bulked sporophytes. As a traditional seedling-raising method, a collection of mature sporophytes are intensively washed with high pressure seawater (A), seawater (B) and flowing seawater (C) successfully. These sporophytes are briefly air dried and then sunk in seawater, letting zoospores release (D). After cleaning, the coir rope curtains (E) are sunk into zoospore-containing seawater, letting zoospores adhere to the curtains. These curtains are arrayed at the bottom of cement pool arrays where young sporophytes develop (F). In order to delete weak sporophytes and unwanted organisms (*e.g.* some microalgae), the curtains are washed with high pressure seawater frequently (G). The seedlings grow indoor in sand filtrated, UV sterilized and temperature constant (8℃) seawater across the whole summer when seawater is ≥20℃, lasting about 3 months (H). The seedlings are temporarily cultured in open sea, transplanted when they are about 20 cm in length (I), cultured and harvested as are done for hybrids (J).

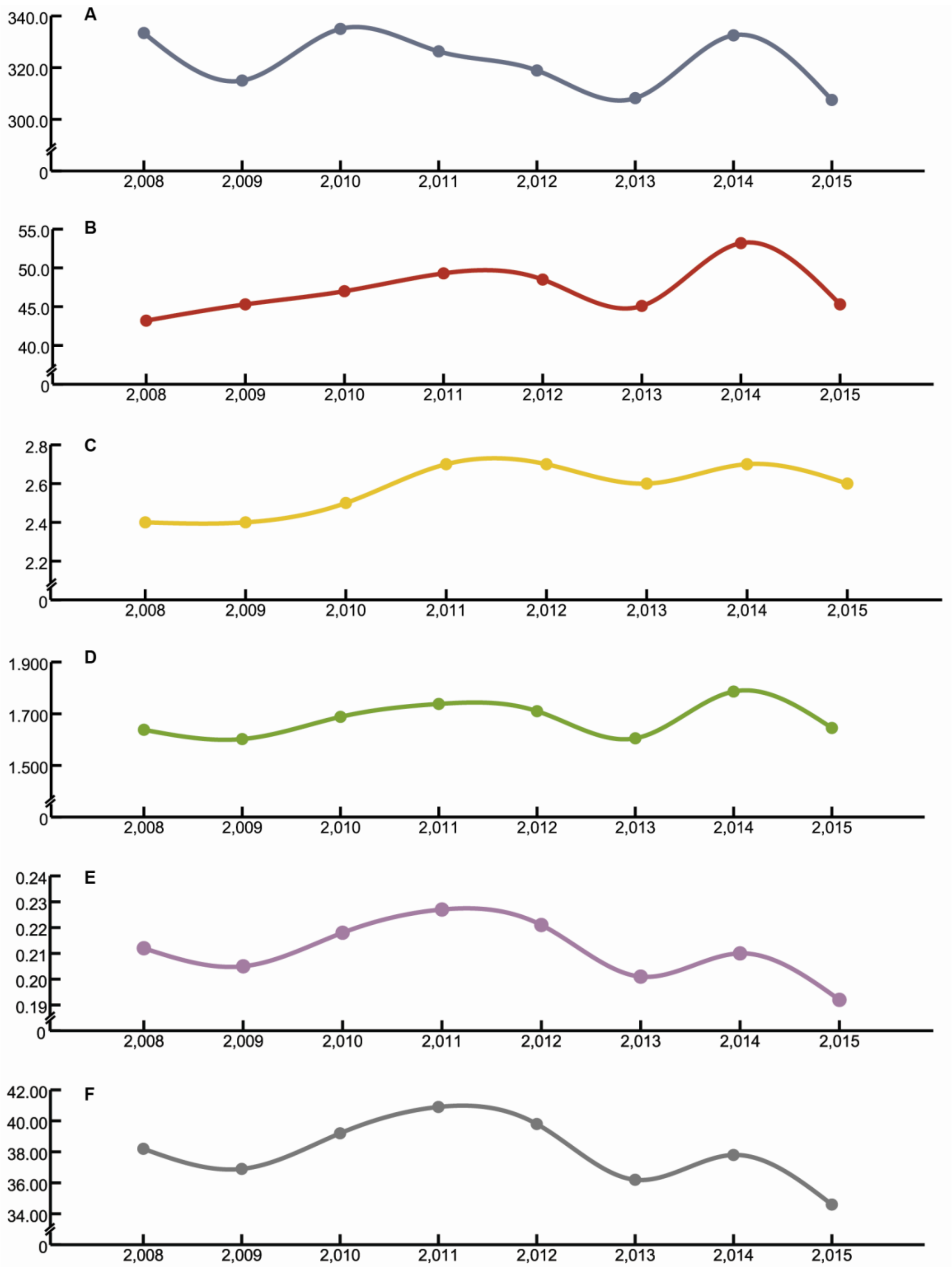

Supplementary Figure 4 The performance of Dongfang no.7 traits including blade length (A), width (B), thickness (C), fresh weight (D) and air dry weight (E) and yield (F) derived from air dry weight by multiplying the dry weight with 180,000.

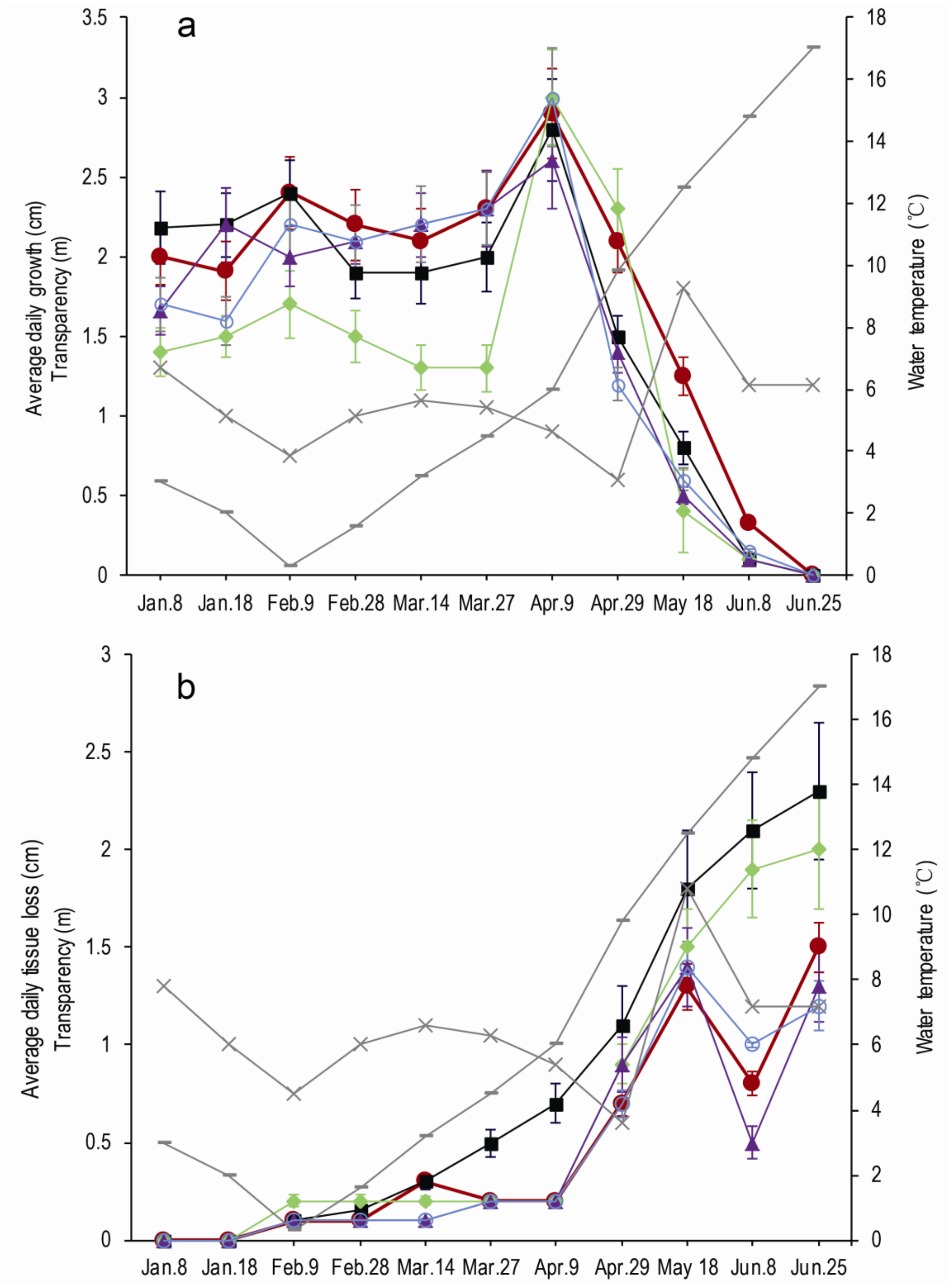

Supplementary Figure 5 Average daily growth of blade (centimeters) (a) and average daily tissue loss (centimeters) (b), seawater transparency (meters) and temperature (℃). Red line dotted with solid red circle, Dongfang no. 7; black line interrupted by black squares, Control 1; green line interrupted by green rhombuses, Control 2; purple line interrupted by triangles, Korean kelp; blue line interrupted by empty circles, Kuanbao haidai; grey line interrupted by crosses, Transparency; grey line interrupted by dashes, Seawater temperature. Average daily growth of blade (a), average tissue loss (b) shares the left vertical axis with seawater transparency.

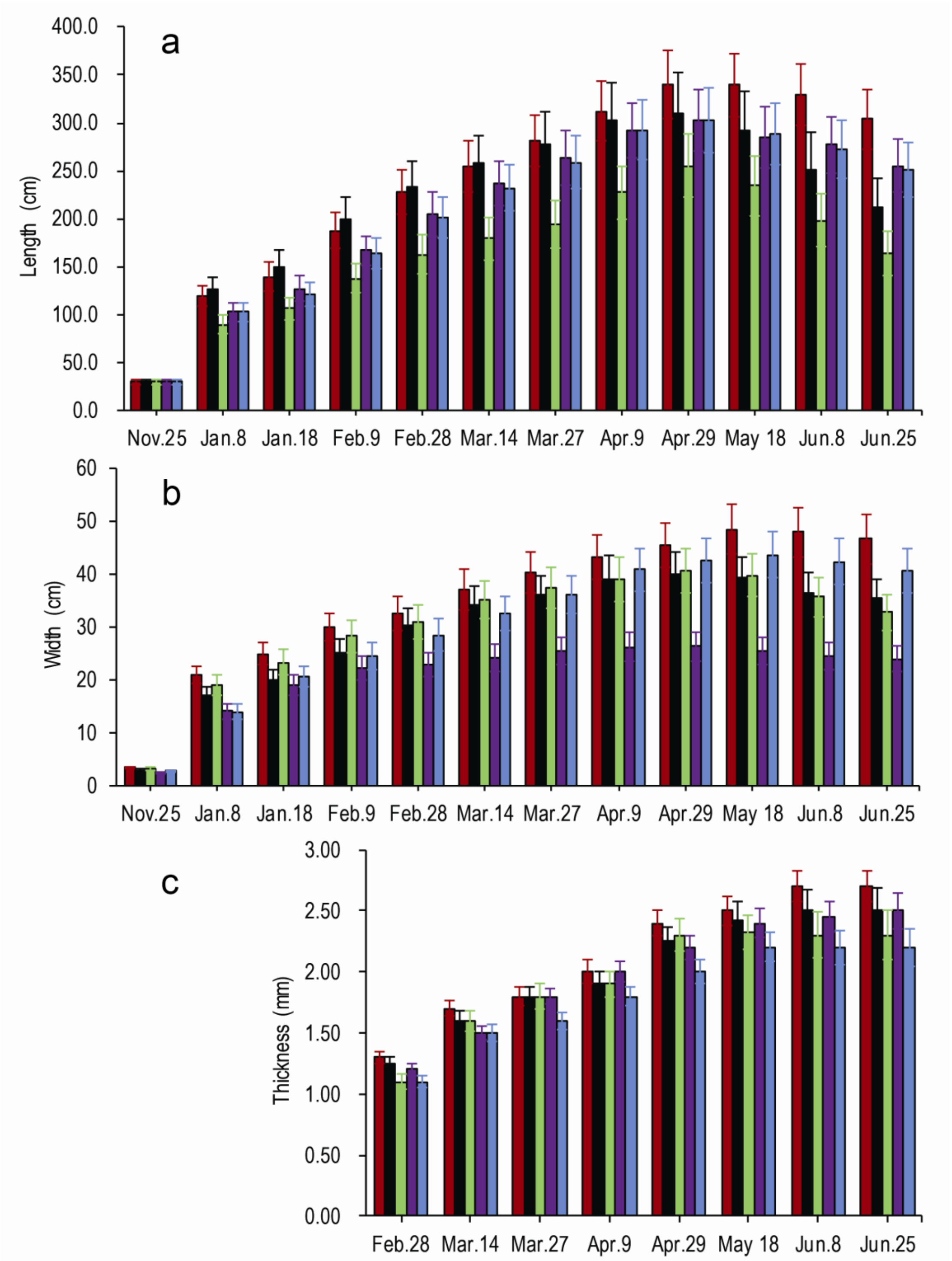

Supplementary Figure 6 The change in blade length (a), width (b) and thickness (c). Red bar, Dongfang no.7; black bar, Control 1; green bar, Control 2; purple bar, Korean kelp; blue bar, Kuanbao haidai.

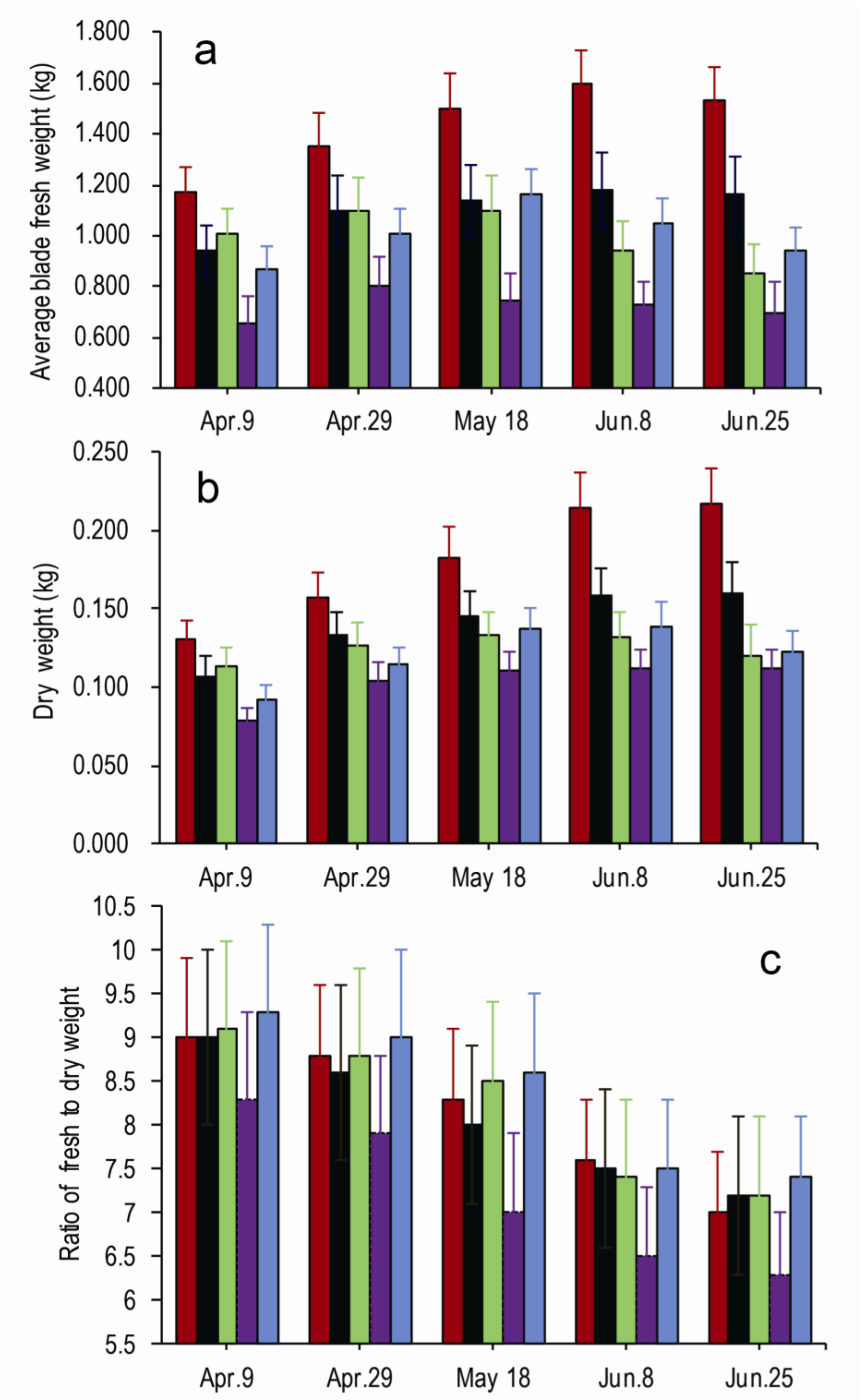

Supplementary Figure 7 Average blade fresh weight (a), average blade dry weight (b) and ratio of fresh to dry weight (c). Red bar, Dongfang no.7; black bar, Control 1; green bar, Control 2; purple bar, Korean kelp; blue bar, Kuanbao haidai.
